# Evaluation of Methyl-Binding Domain Based Enrichment Approaches Revisited

**DOI:** 10.1371/journal.pone.0132205

**Published:** 2015-07-15

**Authors:** Karolina A. Aberg, Linying Xie, Robin F. Chan, Min Zhao, Ashutosh K. Pandey, Gaurav Kumar, Shaunna L. Clark, Edwin J. C. G. van den Oord

**Affiliations:** 1 Center for Biomarker Research and Precision Medicine, Virginia Commonwealth University, Richmond, VA, United States of America; 2 Center for Integrative and Translational Genomics and Department of Anatomy and Neurobiology, University of Tennessee Health Science Center, Memphis, TN, United States of America; University of Bonn, Institut of experimental hematology and transfusion medicine, GERMANY

## Abstract

Methyl-binding domain (MBD) enrichment followed by deep sequencing (MBD-seq), is a robust and cost efficient approach for methylome-wide association studies (MWAS). MBD-seq has been demonstrated to be capable of identifying differentially methylated regions, detecting previously reported robust associations and producing findings that replicate with other technologies such as targeted pyrosequencing of bisulfite converted DNA. There are several kits commercially available that can be used for MBD enrichment. Our previous work has involved MethylMiner (Life Technologies, Foster City, CA, USA) that we chose after careful investigation of its properties. However, in a recent evaluation of five commercially available MBD-enrichment kits the performance of the MethylMiner was deemed poor. Given our positive experience with MethylMiner, we were surprised by this report. In an attempt to reproduce these findings we here have performed a direct comparison of MethylMiner with MethylCap (Diagenode Inc, Denville, NJ, USA), the best performing kit in that study. We find that both MethylMiner and MethylCap are two well performing MBD-enrichment kits. However, MethylMiner shows somewhat better enrichment efficiency and lower levels of background “noise”. In addition, for the purpose of MWAS where we want to investigate the majority of CpGs, we find MethylMiner to be superior as it allows tailoring the enrichment to the regions where most CpGs are located. Using targeted bisulfite sequencing we confirmed that sites where methylation was detected by either MethylMiner or by MethylCap indeed were methylated.

## Introduction

The methylation of DNA cytosine residues at the carbon-5 position is a common epigenetic modification. In mammalian somatic tissues this modification is most often, although not exclusively, found in the context of CpG sequence. Methylation at critical sites can suppress transcription [1] but more recent studies have also associated methylation with increased expression levels [2]. Investigations of DNA methylation marks provide a promising complement to genetics studies of sequence variation as it can, for example, link genetic and environmental effects.

The most comprehensive approach to study genome-wide DNA methylation is whole genome bisulfite sequencing (WGBS), where unmethylated cytosines are converted to uracil [3] and the methylation status of each site is determined after deep sequencing. However, due to the combination of high costs of sequencing entire genomes and the large numbers of samples required for adequate statistical power, this approach is currently not economically feasible for methylome-wide association studies (MWAS) [4].

We have found that methyl-binding domain (MBD) enrichment followed by sequencing (MBD-seq), is a robust and cost efficient approach for MWAS [5]. With this approach, we first fragment the DNA and then aim to extract only the methylated fragments using a protein with high affinity for methylated CpGs [6]. This is more cost-effective than WGBS as only the methylation-enriched DNA portion is used for sequencing [6–8]. MBD-seq has already been demonstrated to be sensitive and capable of identifying differentially methylated regions [6, 8–11], detecting previously reported robust associations [12] and producing findings that replicate when using “gold standard” technologies such as targeted pyrosequencing of bisulfite converted DNA [13].

There are several kits commercially available that can be used for MBD enrichment. Our previous work has involved MethylMiner (Life Technologies, Foster City, CA, USA) [5, 13]. Prior to using this kit, we carefully investigated its properties. These evaluations suggested that the background noise level of MethylMiner is relatively low, (the ratio between the median coverage for a CpG, i.e., the methylation signal, versus a non-CpG, i.e., the background noise, was 40.6) and that a good enrichment of methylated fragments can be obtained [5]. Another appealing feature is that methylome-wide coverage can be optimized by using a protocol version where the enriched fraction is eluted with a low salt concentration [5].

Given our positive experience with MethylMiner, we were surprised to learn that in a recent evaluation of five commercially available MBD-enrichment kits [14] the performance of the MethylMiner was deemed poor. In an attempt to reproduce these findings we here have performed a direct comparison of MethylMiner with MethylCap (Diagenode Inc, Denville, NJ, USA), which in the previous evaluation was considered the most optimal MBD enrichment kit [14].

Both MethylMiner and MethylCap use proteins for enrichment of the CpG methylated fraction of the genome. MethylMiner uses a biotin conjugated methyl-CpG binding domain (MBD) of human Methyl-CpG-binding domain protein 2 (MBD2). The biotin epitope of the MethylMiner protein allows for pulldown of captured DNA with paramagnetic streptavidin beads. Exploiting the sensitivity of protein-DNA interactions to increasing salt concentrations, MethylMiner allows the user to elute DNA fractions based on the density of methylated CpGs. Instead of MBD2 MethylCap uses a fusion protein of the MBD of human methyl-CpG-binding protein 2 (MECP2) and Glutathione-*S*-transferase (GST). The GST domain of the MethylCap fusion protein binds then to glutathione coated paramagnetic beads for extraction.

To compare the two kits we performed methylome wide profiling for each kit using the same two mice, in duplicates. To further evaluate our findings, a number of randomly selected CpGs from sites where we observed differences were validated in the same samples, in duplicate, using targeted bisulfite pyrosequencing. Furthermore, to evaluate whether the methylome-wide coverage of MethylCap could target regions with different CpG densities a third mouse was investigated with MethylCap using the lowest and the highest elution buffer concentrations recommended. This data was compared with previously unpublished data from a similar evaluation of MethylMiner conducted in two human samples.

## Materials and Methods

### Preparation of DNA

Adult DBA2/J male mice from Jackson Laboratories (Bar Harbor, ME, United States) were housed 4 per cage on a 12-h/12-h light/dark cycle in an AAALAC-accredited animal facility with continuous access to standard chow (Harlan Teklad #7912, Madison WI) and water. At the time of tissue extraction cervical dislocation was performed and striatum brain tissue was extracted from each mouse. Tissue was frozen in liquid nitrogen and stored in -80°C until DNA extraction. All procedures for housing and handling the animals were approved by the Virginia Commonwealth University Institutional Animal Care and Use Committee under protocol number AM10332 and carried out in strict accordance with the recommendations in the National Institute of Health Guide for the Care and Use of Laboratory Animals (NIH Publications No. 80–23, 1996).

Genomic DNA was extracted from brain tissue using the Gentra Puregene Blood/Tissue Kit (Qiagen, Valencia, CA) following the vendor’s instructions. The DNA was fragmented into a median length of 180 base pairs using ultrasonication with the Covaris S2 instrument (Covaris, Woburn, MA, USA). The fragmentation was performed in frequency sweeping mode for 6 cycles using the following conditions: duty cycle 10%, intensity 5, and 100 cycles per burst during 60 seconds. The water temperature limit was set to 15°C and the water temperature was maintained at 5°C. For each mouse, DNA was fragmented in amounts of 3 μg and pooled prior to further procedures. Thus, the fragment length distribution was identical for all conditions investigated from the same mouse. The length of the fragmented DNA was determined with the Agilent High Sensitivity Kit on an Agilent Bioanalyzer 2100 (Agilent Technologies, Santa Clara, CA, USA) and the DNA was quantified with the NanoDrop ND-1000 spectrophotometer (Thermo Fisher Scientific, Wilmington, DE).

### Methylation enrichment

The amount of starting material for each methylation enrichment reaction was 1.5 μg of DNA for both MethylMiner and MethylCap. The standard MethylCap protocol specifies using up to 1.0 μg of genomic DNA as starting material. Therefore, we followed advice from the vendor and adjusted the reagent volumes accordingly to accommodate for the 1.5 μg used in this study. Except for the minor adjustment made to obtain comparable amounts of input material, enrichment for both kits was performed according to standard protocols with the lowest salt concentration recommended for elution (0.5M NaCl and Low Elution Buffer– 0.5M, for MethylMiner and MethylCap, respectively). As summarized in **Table 1**, the two capture methods were performed for the same two samples. To study the reproducibility of these assays we also used technical duplicates. To study the effects of altered salt concentrations for elution we also performed enrichments with MethylCap in a third mouse. For these enrichments we used the lowest (Low Elution Buffer– 0.5M) and the highest (High Elution Buffer—0.8M) salt concentrations recommended for MethylCap.

**Table 1:** Overview of study design for the MethylMiner versus MethylCap comparison and number of reads in each condition. Captured DNA (ng) indicates the amount of DNA that was captured in the MBD enrichment; % Mapped Reads are percent of the Total Reads that successfully aligned and % Used Reads are percent of the Total Reads that successfully aligned and remained after rigorous quality control.

Individual	Technical Duplicate	MBD-capture Kit	Captured DNA (ng)	Total Reads	% Mapped Reads	% Used Reads
A	1	MethylMiner	284	32,786,373	71.9	28.2
A	2	MethylMiner	273	43,543,509	71.9	25.1
A	1	MethylCap	77	32,213,938	72.9	29.9
A	2	MethylCap	85	23,883,650	74.0	30.3
B	1	MethylMiner	317	48,397,266	72.2	32.2
B	2	MethylMiner	312	42,712,240	70.5	34.0
B	1	MethylCap	115	31,148,759	74.0	34.9
B	2	MethylCap	106	29,959,582	72.9	31.5

### Library preparation and sequencing

The methylation-enriched DNA from MethylMiner (mean = 296.5 ng, SD = 21.4) and MethylCap (mean = 95.8 ng, SD = 17.7), respectively, was used as input materials for barcoded fragment libraries for SOLiD5500xl Wildfire deep sequencing. This library construction protocol allows for 10 ng to 5 μg of DNA to be used as starting material. The protocol includes size selection of fragments of optimal length for the sequencing. This size selection prevents any unexpected long or short fragments from being included in the sequencing. Each library was sequenced using single-end chemistry at 50 bp read length. After deleting reads with more than 2 missing color calls, for the eight samples in the primary comparison, we obtained on average 35.6 million reads per sample (SD = 8.3 million).

### Alignment, quality control, and coverage calculations

The sequenced reads were aligned to the DBA2/J (D2) specific reference genome[15] (see supplementary materials for details about the reference genome, S1 Text) using BioScope 1.2 (Life Technologies). The mean percentage of mapped reads was 71.6% (SD = 0.8) for MethylMiner and 73.5% (SD = 0.6) for MethylCap. We have previously developed an analysis pipeline for MBD-seq that includes rigorous quality control for methylome-wide investigations[5]. This quality control includes exclusion of duplicate reads (i.e. reads starting at the same location) likely to be caused by amplification artifacts, and exclusion of low-quality multi reads (i.e. reads that align equally well to a large number of locations in the genome). After exclusion of low quality multi reads and duplicate reads 39.6% (sd = 3.5) and 45.1% (sd = 1.8) of the mapped reads were retained for MethylMiner and MethylCap, respectively. It should be noted that the percentage of reads remaining after quality control (i.e. used reads) are slightly lower than what we typically see when working with more established reference genomes such as the human genome and the commonly used C57BL6 mouse genome.

The MBD proteins have high affinity to methylated CpGs but not to methylated Cs (CpH) outside the context of CpGs. Therefore, we considered only the 20.3 million CpG sites in the autosomal mouse reference genome for our analysis. Methylation measurements were obtained by estimating how many fragments covered each CpG, which gives a more precise measurement than, for example, counting the reads or extended reads (e.g. to the expected fragment length) covering each CpG[16]. The method is described elsewhere in detail[16]. In summary, using the distribution of reads around isolated CpGs, we first obtained non-parametric estimates of the fragment size distribution for each sample. The sample-specific estimated fragment size distribution was then used to calculate the probability for each read that the fragment it is tagging covers the CpG under consideration. Coverage estimates for each CpG were then calculated for each subject by taking the sum of the probabilities that all fragments in its neighborhood cover the CpG. Coverage estimates obtained with this method show very high correlations (R = 0.999) with estimates obtained using paired-end libraries where fragment size distributions are known[16]. The coverage calculations are standardized on the total number of used reads for each sample.

### Enrichment efficiency

Reads aligned to regions that lack CpGs will yield an estimate of the non-specific binding (i.e. “noise”), which can be used to estimate the enrichment efficiency of each kit. Here we used the 95^th^ percentile of the coverage estimates at non-CpGs (defined here as sites located at least 400 bp from any known CpG) as the threshold for “noise”. Thus, sites with a coverage estimate that exceeds the threshold for noise are considered methylated.

### Percent of CpG coverage

For optimal methylome-wide coverage it is important that the sequenced fragments cover the vast majority of CpGs. To study this, we first calculated the local CpG density (also called coupling factor[17]) by counting the number of CpGs within 100 bp on either side of a target CpG. Next, we calculated a percent of CpG coverage measure denoted as $C_{d}^{s}$, that roughly speaking can be interpreted as the portion of all fragments covering sites with a local CpG density of d; as follows:

$$C_{d}^{s}=\frac{N_{d}\left(C_{d}-C_{95}^{0}\right)}{\sum_{d=1}^{k}N_{d}\left(C_{d}-C_{95}^{0}\right)}$$ (1)

Thus, $C_{d}^{s}$ is the standardized version of *C*_*d*,_ with *N*_*d*_ being the number of sites in the genome that have local CpG density *d*, *C*_*d*_ = *Mean*(*C*|*D* = *d*) or the (conditional) mean coverage *C* of sites with local CpG density *d*, $C_{95}^{0}$ is the threshold for background noise for the tested kit estimated using the 95^th^ percentile of non-CpG coverage, and *k* the maximum CpG density.

### Validation with targeted bisulfite pyrosequencing

Differences between MethylMiner and MethylCap were further studied with an independent technology. This validation was performed using aliquots of the same DNA samples as were used for the MBD-seq analysis. Genomic DNA was bisulfite converted using Epitect 96 (QIAGEN, Germantown, MD). For each assay, the bisulfite converted DNA was used as input material for the PyroMark PCR in a total reaction volume of 12.5 μl. In the PyroMark pyrosequencing reactions (QIAGEN, Germantown, MD) 2.5 μl of the amplicons were used. With the exception that we used universal primers[18, 19], all procedures were conducted according to the standard protocols provided by the vendor. Each pyrosequencing assay was designed using the PyroMark Assay Design software (QIAGEN, Germantown, MD). All DNA samples were investigated in duplicates.

In addition to the investigated samples, each assay included analysis of four different negative controls and three standards with known methylation levels. The negative pyrosequencing controls consisted of 1) PCR product conducted without DNA, replaced with H2O, 2) pyrosequencing reaction without amplicon and PCR primers, 3) pyrosequencing reaction without amplicon but with the biotinlylated PCR primer included, 4) pyrosequencing reaction without the sequencing primer and without amplicon but with the biotinlylated PCR primer included. The known standards included three DNA samples in duplicates with known methylation levels (0%, 50% and 100% methylation). The methylation standards were created using fully methylated control DNA, following the recommended protocol for “Methylation of Genomic DNA” (New England BioLab, Ipswich, MA) and unmethylated control DNA, using the REPLI-g amplified DNA protocol (QIAGEN, Germantown, MD) created from the same DBA2/J mouse strain.

All assays passed careful quality control. First, all negative controls were checked to be truly negative. Next, the known standards were checked to have values in the approximate range of the expected methylation level. Similar to what we previously have seen in other studies, we occasionally noticed that, although values were linear, the methylation levels for the known standards were shifted for different CpG sites within the same assay or consistently slightly shifted for an entire assay. These assays were still evaluated as good quality assays, as the shifts are likely to be true observations, caused by slightly variable methylation levels in the synthetically created fully methylated and unmethylated DNA. In addition, the pyrograms of the known standards and the DNA samples to be investigated were checked to match the reference sequence pattern and the peak heights were checked to match the histogram of expected values. Finally, to ensure that the bisulfite conversion was sufficient we included bisulfite conversion controls in all assay where possible.

## Results

**Table 1**shows the amount of DNA captured, the number of total reads, aligned reads, and reads that remained after quality control. When comparing the estimated coverage for each CpG site from our technical duplicates we observed a Pearson correlation of 0.838 and 0.851 for MethylMiner and 0.867 and 0.883 for MethylCap. These correlations indicate reproducibility, and suggest that the enrichment was successful for both kits. Furthermore, the relatively high correlations between the technical duplicates suggest that the performance of the enrichment was not negatively affected by using a slightly increased amount of DNA as starting material.

**Fig 1:**
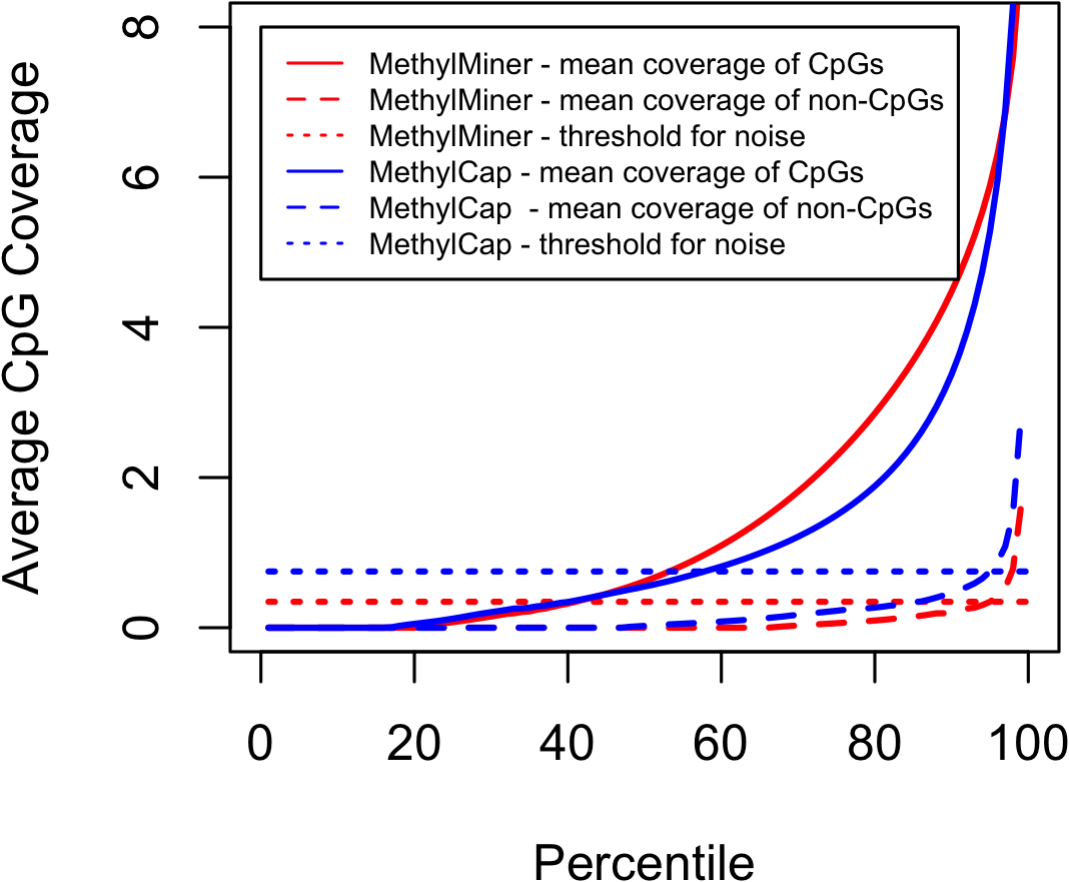
Methylation signals and levels of background noise. The mean coverage of CpGs and non-CpGs from MethylMiner and MethylCap, respectively are shown. The horizontal lines indicate the threshold for background noise as determined by the 95^th^ percentile of the estimated coverage of the non-CpGs for MethylMiner and MethylCap, respectively.

**Fig 1**shows the percentiles of the average estimated coverage for CpGs and non-CpGs, respectively. Results show that the coverage for non-CpGs is generally lower for MethylMiner than for MethylCap, whereas coverage for CpGs is generally higher for MethylMiner than for MethylCap. Furthermore, for MethylMiner 59% of the CpGs show coverage estimates that are higher than the threshold for background noise (i.e., can be considered methylated), whereas for MethylCap the corresponding value is 42%. Thus, MethylMiner shows less noise and better enrichment than MethylCap.

In total, the MethylMiner kit detected 12,026,178 methylated sites and the MethylCap kit detected 8,601,775 methylated sites. The overlap between the two kits was 7,888,063 sites. **S1 Table** gives the number of methylated sites uniquely detected by each of the two kits as well as the number of sited detected by both kits for each CpG density.

**Fig 2:**
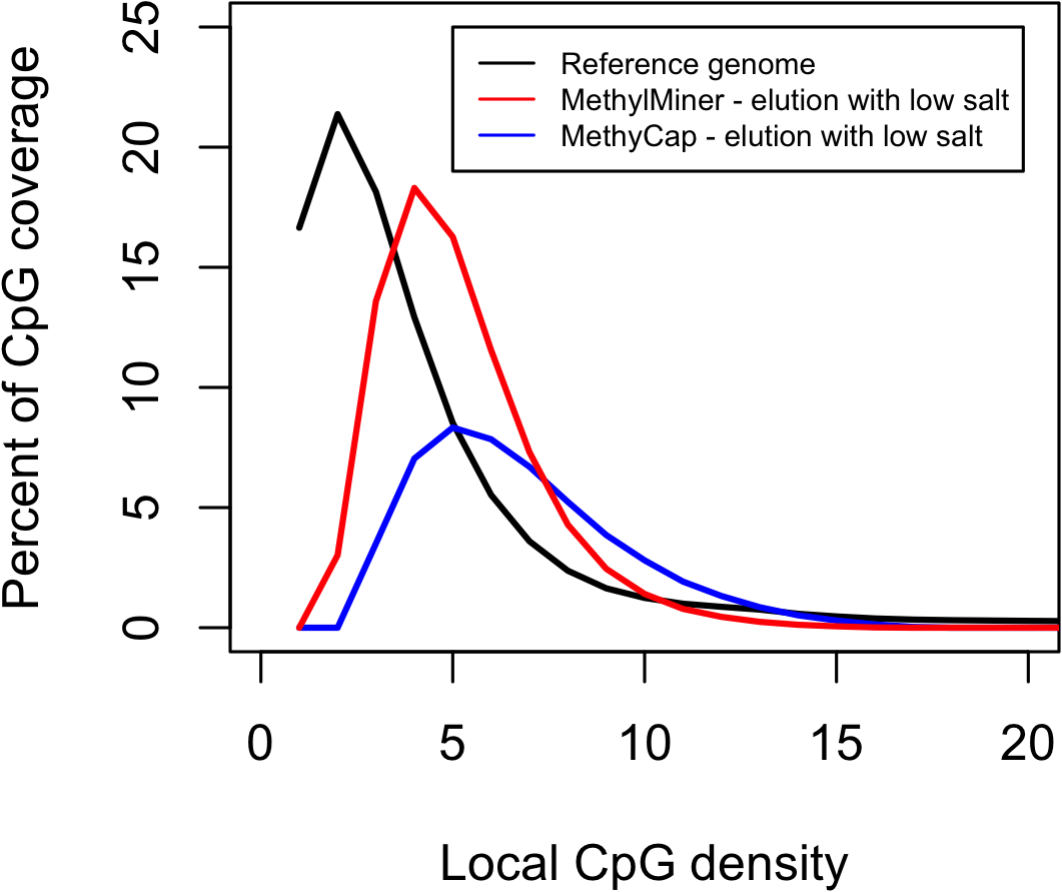
The overlap of MethylMiner and MethylCap with local CpG density. The local CpG density is plotted against the percentage CpG coverage. The distribution of the local CpG density in the reference sequence is the highest outside of CpG-rich regions. Better coverage of the regions where the majority of CpGs occur (in the range of 1–7 CpGs) is obtained with MethylMiner than with MethylCap.

In **Fig 2**the local CpG density is plotted against the percent CpG coverage measure $C_{d}^{s}$, for MethylMiner and MethylCap, respectively. To evaluate if the percent of CpG coverage measures were significantly different between MethylMiner and MethylCap we randomly selected 10,000 independent sites for each density (a maximum of 1 site was selected per 400bp to avoid correlated observation that would violate assumptions underlying our statistical test) from across the genome and compared their methylation status (methylated vs. unmethylated) using a chi square test. Using a stringent threshold of α = 0.01 that was Bonferroni corrected for 20 tests (one for each CpG density) the threshold for declaring significance was 5.00x10^-04^. For each of the CpG densities from 1 to 11 our results indicate significant differences between the number of methylated CpGs detected with MethylMiner as compared to MethylCap (p ranging from 9.66x10^-07^ to <5.98x10^-283^). In contrast, when studying each of the CpG densities between 12 and 20 CpGs we did not observe significant differences between the two kits.

Furthermore, our results show that the distribution of CpGs in the genome more closely resembles the distribution of the standardized coverage as observed for MethylMiner than for MethylCap. Because MethylMiner overlaps much better with the distribution of CpGs across the genome, it provides better coverage and therefore a more complete investigation of the CpG methylome across all CpG densities.

### Validation of discrepancies using targeted bisulfite pyrosequencing

In the most optimal scenario we would validate all detected discrepancies using an independent technology, such as e.g. whole genome bisulfite sequencing or similar techniques. In the absence of funding for such a comprehensive validation we have instead preformed a targeted validation of selected sites. We designed pyrosequencing assays for ten randomly selected sites in the CpG density range of 1–2, that were considered methylated by MethylMiner but were methylation was not detected using MethylCap, and for three sites that were considered methylated by MethylCap but where methylation was not detected by MethylMiner. In addition, we also designed pyrosequencing assays for ten randomly selected sites in the CpG density range of 9–11, that were considered methylated using MethylCap but were methylation was not detected using MethylMiner.

Due to challenges in the DNA sequences, in particular in regions with low sequence complexity due to high GC content, high quality assays could not be designed for all selected loci. Therefore, after *in silico* evaluation eight, three and six assays were performed for the three selection categories described above, respectively. The primers used for the assays and the coordinates for the investigated sites are given in **S2 and S3 Tables**.

When performing validation of sites detected as methylated by MethylMiner but not by MethylCap, using targeted bisulfite pyrosequencing, we found that all sites had a high level of methylation. The methylation levels for the two investigated samples for the eight investigated sites ranged from 68.8% to 98.1% (see **S4 Table** for sample specific methylation levels for each locus). Thus, for all investigated sites the validation results indicated that MethylMiner correctly detected the presence of methylation at these sites. Similarly, when performing validation of sites with low CpG density detected as methylated by MethylCap but not by MethylMiner, we also observed a relatively high level of methylation ranging from 52.8% to 95.7% (**S5 Table**). In contrast, for sites in high CpG density regions that were detected by MethylCap but not by MethylMiner the methylation levels were relatively low, ranging from 10.9% to 32.6% (**S6 Table**). Thus, the validation results indicated that also MethylCap correctly detected the presence of methylation at these sites.

### Targeting the CpG density of interest

**Fig 3:**
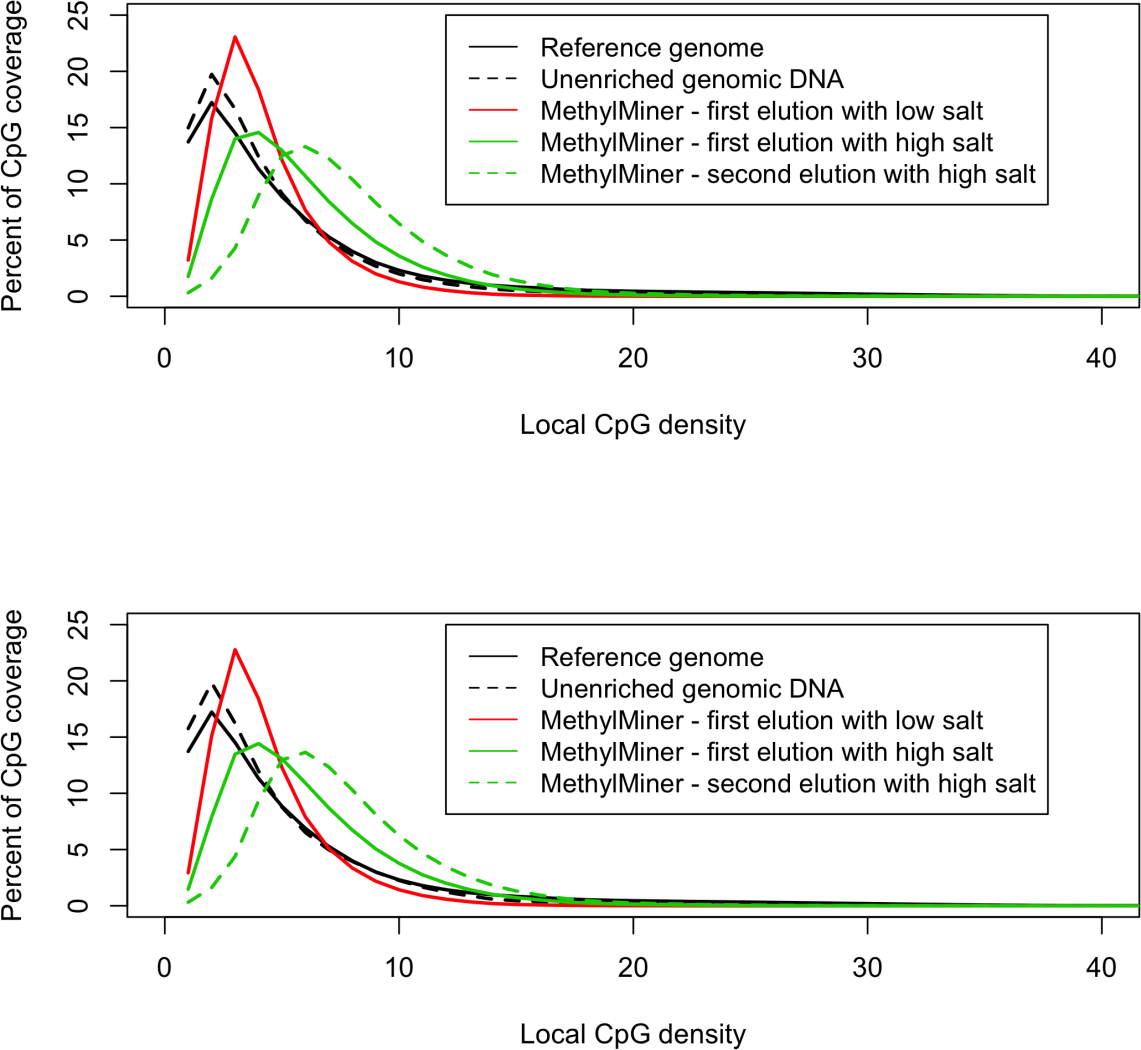
The effect of altered salt concentrations for elutions in MethylMiner. The shown data was generated as part of our recent methylome-wide investigations in humans[5]. The local CpG density is plotted against the percentage CpG coverage for two randomly selected individuals in the upper and lower graph, respectively. As expected, the unenriched genomic DNA closely follows the distribution of the CpG density in the reference genome. Best coverage of the regions where the majority of CpGs occur (in the range of 1–7 CpGs) is obtained by a single MethylMiner elution using a low salt (0.5M NaCl) buffer. Using a single elution with a higher salt (2.0M NaCl) concentration moves the distribution to regions with higher CpG density. If performing a double elution where the low salt elution is discarded and only the 2^nd^ fraction eluted with the high salt buffer is investigated, the distribution is shifted even further to the right excluding regions with low local CpG density and focusing mainly in regions with high CpG density.

According to the manufacturer the distribution of the methylated fraction enriched for by MethylMiner can be shifted towards CpG richer regions by increasing the salt concentration of the elution buffer (see the application note for MethylMiner [20]). To validate this claim we previously performed an evaluation using human DNA that were part of our recent methylome-wide investigations[5]. **Fig 3**shows previously unpublished data from this comparison of two human samples. The percentage CpG coverage measure $C_{d}^{s}$ is plotted against the local CpG density. Best coverage of the regions where the majority of CpGs are (i.e. the range of 1–7 CpGs) is obtained by a single MethylMiner elution using a low salt (0.5M NaCl) buffer. Using a single elution with a higher salt (2.0M NaCl) concentration moves the percentage of CpG coverage distribution in the direction of CpG dense regions. However, as relatively few CpGs are located in dense regions, the overall coverage of the methylome is reduced with a high salt elution. This effect is even further enhanced if a double elution is performed where the low salt elution is discarded and only the second elution, performed with the higher salt concentration, is sequenced. Thus, these results suggest that using a low salt concentration will give the most comprehensive coverage of the CpG methylome that can be achieved with the MethylMiner kit.

To evaluate if the methylome-wide coverage of MethylCap could target regions with different CpG densities we in this study performed a similar evaluation in a third mouse. For this additional mouse sample MethylCap was performed using the lowest and the highest elution buffer concentrations recommended by the vendor.

**Fig 4:**
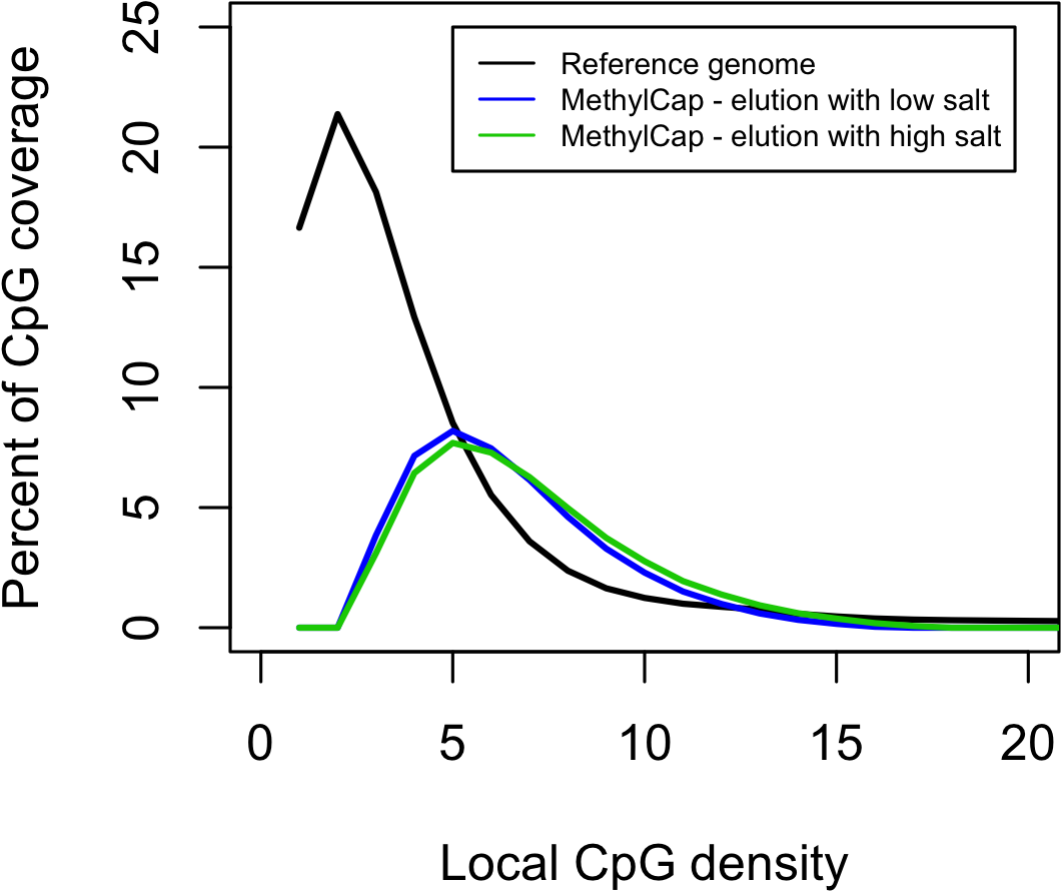
The effect of altered salt concentrations for elutions in MethylCap. The local CpG density is plotted against and the percentage CpG coverage. The distribution of the local CpG density in the reference genome is shown. The distribution of CpG coverage for MethylCap is shown when using a low salt elution buffer and when using a high salt elution buffer.

**Fig 4**shows that when the concentration of the elution buffer was altered for MethylCap we did not observe a notable shift of the percent CpG coverage. Therefore, under the conditions used in this study, the elution conditions for MethylCap cannot be easily adapted to improve enrichment for the CpG densities where the majority of CpGs are found.

## Discussion

We found that MethylMiner and MethylCap both yield sufficient amounts of methylation enriched DNA for construction of high quality sequencing libraries. The total number of reads, percentage of mapped reads and the percentage of reads passing quality control were similar for the two enrichment kits. We also found that the sample correlations of the estimated methylation coverage between technical duplicates (0.838 and 0.851 for MethylMiner; 0.867 and 0.883 for MethylCap were similar for the two kits. Although these correlations suggest some noise, they also indicate a consistency in the enrichment procedure for both kits. Thus, the MBD-seq protocols were successful for both kits and the results were comparable.

We also observed that with MethylMiner the coverage of CpGs was higher and background “noise” lower. These results are in line with previous studies suggesting that the MBD2 protein, which is used in MethylMiner, has a stronger affinity for methylated CpGs vs. unmethylated DNA than what has been observed for the MeCP2 protein[21, 22]. The better detection of CpGs from CpG poor regions and the better signal to noise ratio translate to being able to detect more CpGs. Indeed, MethylMiner detected 59% and MethylCap 42% methylated CpGs.

The 59% methylated CpGs in mouse brain detected with MethylMiner is in line with what recently has been reported. Using whole-genome bisulfite sequencing Lister et al. found ~60–70% methylated CpGs in frontal cortex from mice of age 4 weeks to 22 months[23]. Although, the most comprehensive comparison would include whole-genome bisulfite sequencing of the same samples as were used in the present study, there are no indications that the percentage of sites detected in our current investigation is limited by the specificity of MethylMiner but rather that it reflects the methylome of the investigated mice or the methodological set up used, which is not directly related to MethylMiner. For example, for all MBD-seq approaches the specificity is a combination of the sensitivity of the kit and the number of used sequence reads. Thus, if the enrichment kit has a high sensitivity but the number of used reads is low, the specificity may still be limited and regions where methylation is moderate may not be covered. The previous study in humans[5] included more than twice as many reads as we used in the present study. This difference, which results in altered specificity, may partly contribute to the different percentage of methylated CpGs observed in this study and in the previous human study.

Our evaluation of MethylMiner and MethylCap came to a dramatically different conclusion compared to the previous evaluation of these kits, which reported that MethylMiner lacks sensitivity and specificity[14]. In contrast, we found that both MethylMiner and MethylCap were two well performing MBD-enrichment kits, where MethylMiner performed better than MethylCap in terms of enrichment efficiency and gave a better coverage of CpG poor regions where most of the CpGs are located.

It should be noted that there are a number of differences between the present work and the study previously published by De Meyer et al. For example, the previous work was exclusively conducted in human cell lines while the present work is conducted in brain tissue from the DBA2/J mouse strain and from human blood samples. However, given that the two DNA binding proteins used by MethylMiner and MethylCap both bind to the same type of DNA methylation (methylated CpG sites) discrepancies in performances between the two kits are unlikely to be caused by the origin of the DNA. Other differences between the two studies included a number of lab technical variables such as elution conditions of the captured methylated DNA and the sequencing chemistry/platforms used. In addition to using MethylMiner in combination with sequencing on SOLiD platforms we have previously also successfully used MethylMiner with human DNA in combination with other sequencing platforms including both MiSeq (Illumina) and NextSeq500 (Illumina) (unpublished data). Thus, while it is possible the differences in design and set up between our present study and the previous study by De Meyer et al. may have some effects on the results it is unlikely that any, or all, of these factors would have caused the MethylMiner enrichment to completely fail in the way that was reported by De Meyer et al [14].

A more critical difference between the present and the previous study by De Meyer et al. may be the amount of starting material used for the enrichment protocols and whether the amount of DNA in the enriched fraction is sufficient for high quality library construction. We have previously shown that the amount of starting material is of importance to sufficiently extract the methylated fraction of the genome and subsequently create a complex sequencing library [24]. We experienced that optimal quality MBD-seq analysis with MethylMiner and the sequencing protocols described in the current work was achieved when starting with >500 ng DNA as input material for MethylMiner. When using the standard MethylMiner protocol and decreasing the amount of starting material to 250 ng we observed somewhat decreased performance[24] and when further decreasing the amount of starting material we repeatedly observed a much lower signal to noise ratio (unpublished data). Although it is unlikely that the procedure would completely fail by using slightly less than 250 ng starting material it should be encouraged to use a modified protocol, provided by the vendor (Life Technologies, Foster City, CA, USA), which is optimized for sub-micrograms of starting material.

The amount of starting material used for the methylation enrichment in the previous evaluation by De Meyer et al. was 200 ng DNA and the amount enriched material used for the subsequent library construction ranged from 1.75 ng to 48.30 ng. While there are library construction protocols that have been optimized to successfully handle low amounts of input material, the previous evaluation[14] used a protocol that typically calls for 1–5 ug of DNA as starting material. Thus, the poor performance of MethylMiner reported previously may not reflect MBD-enrichment capacity, but may be caused by problems in the library construction process.

## Conclusions

We find that both MethylMiner and MethylCap are two well performing MBD-enrichment kits. However, MethylMiner show somewhat better enrichment efficiency and lower levels of background “noise”. In addition, for the purpose of MWAS where we want to investigate the majority of CpGs, we find MethylMiner to be superior as it allows tailoring the enrichment to the regions where most CpGs are located.

## Supporting Information

S1 Table Number of methylated sites detected. (DOCX)

S2 Table Pyrosequencing assay design for sites detected by MethylMiner. (DOCX)

S3 Table Pyrosequencing assay design for sites detected by MethylCap. (DOCX)

S4 Table Methylation detected by MethylMiner for CpG density range 1–3. (DOCX)

S5 Table Methylation detected by MethylCap for CpG density range 1–3. (DOCX)

S6 Table Methylation detected by MethylCap for CpG density range 9–11. (DOCX)

S1 Text Mouse reference genome. (DOCX)
