# Supplementary material for: Evaluation of Methyl-Binding Domain Based Enrichment Approaches Revisited
Source: PLoS One. 2015 Jul 15;10(7):e0132205. doi: 10.1371/journal.pone.0132205 (PMC4503759; doi:10.1371/journal.pone.0132205)
Supplement: S1 Table — (DOCX) [file pone.0132205.s001.docx]

# S1 TABLE. Number of methylated sites detected.

| CpG density | MethylCap only | MethylMiner only | Both kits | Number of site in the genome |
| --- | --- | --- | --- | --- |
| 0 | 246,150 | 294,227 | 247,532 | 3,392,738 |
| 1 | 290,213 | 1,077,988 | 890,419 | 4,355,915 |
| 2 | 89,432 | 1,291,243 | 1,538,630 | 3,695,661 |
| 3 | 20,166 | 751,051 | 1,545,276 | 2,632,731 |
| 4 | 6,259 | 350,313 | 1,219,328 | 1,736,546 |
| 5 | 3,599 | 159,835 | 844,519 | 1,126,133 |
| 6 | 3,393 | 76,987 | 554,290 | 732,920 |
| 7 | 3,656 | 39,760 | 353,054 | 483,234 |
| 8 | 4,285 | 24,071 | 224,814 | 334,000 |
| 9 | 5,370 | 16,722 | 148,901 | 252,532 |
| 10 | 6,215 | 12,082 | 100,301 | 203,478 |
| 11 | 6,999 | 9,996 | 71,442 | 177,318 |
| 12 to 51 | 27,975 | 33,840 | 149,557 | 1,256,404 |
| Sum | 713,712 | 4,138,115 | 7,888,063 | 20,379,610 |
