# Supplementary material for: Evaluation of Methyl-Binding Domain Based Enrichment Approaches Revisited
Source: PLoS One. 2015 Jul 15;10(7):e0132205. doi: 10.1371/journal.pone.0132205 (PMC4503759; doi:10.1371/journal.pone.0132205)
Supplement: S2 Table — (DOCX) [file pone.0132205.s002.docx]

# S2 TABLE. Pyrosequencing assay design for sites detected by MethylMiner.

| Chr | Location (bp) | Strand | Universal primer | PCR primers (forward and reverse primers are given on the top and bottom rows, respectively.) | Sequencing primers |
| --- | --- | --- | --- | --- | --- |
| 3 | 88312855 | + | 1 | GAC GGG ACA CCG CTG ATC GTT TAT TTA GGG AGA ATA TTG TGG GGA GGA A | ATT TTA AAA AAA ATC CTC CA |
|  |  |  |  | CAA ACC ACA CCT ACC TCT ACT TAT |  |
| 4 | 121667807 | + | 2 | CGC CAG GGT TTT CCC AGT CAC GAC AAG GTG GAG ATG AAA TGA TTA TTG G | ACA AAA TCT CCT TCA CTA TA |
|  |  |  |  | CCA CAA CCC CAC TAT TTT ATT TTT CTT TCT |  |
| 7 | 117081770 | - | 2 | AAG GGT GTT GTA TAA AGA GTT GAT | GAT TTA TAT GAT TTA AAA ATT TAG T |
|  |  |  |  | CGC CAG GGT TTT CCC AGT CAC GAC AAT TAA ATC CAA TTA ACT TCC ATC TAT CT |  |
| 9 | 115888715 | + | 1 | TTT GGA TTT GAA GGT GTG TTT TAA GG | GTG TGT TTT AAG GTT ATG T |
|  |  |  |  | GAC GGG ACA CCG CTG ATC GTT TAC ACA TCC CAA ACC TTC TAA ATC T |  |
| 10 | 94733369 | - | 2 | CGC CAG GGT TTT CCC AGT CAC GAC TTG AGT GTG GAT TTT TAG GAT TTA TGT | ACT TCC AAA CCC CAA |
|  |  |  |  | TCC TCA ACT ATT TTC CAC CTT ATT TTC T |  |
| 13 | 41715278 | - | 1 | GAC GGG ACA CCG CTG ATC GTT TAT GAG TTA TTT TTT TAG TTT TTG GTG TGT A | CCA TCT TTA TAC CTA CAA CTT AT |
|  |  |  |  | TCT CTC TAA CCA CCC ATC TTT ATA CC |  |
| 15 | 10987836 | + | 1 | TTT GGG TTT TTT GGT GAG TTG G | GGG TAG TAG AGG TTT AGG |
|  |  |  |  | GAC GGG ACA CCG CTG ATC GTT TAC ATC TAT CTT CCT TTC AAA TAC TAA CAC |  |
| 16 | 57553603 | + | 1 | AGT TGT GAG TTT AGT AGA GAA GGT GTA TT | GGA GGT TAG TTA GTG ATA T |
|  |  |  |  | GAC GGG ACA CCG CTG ATC GTT TAT CAA CTC TTC TCC CTT CCC AAA AAA |  |

Note. Chr is chromosome; 5' Biotin modified universal primer 1 = GAC GGG ACA CCG CTG ATC GTT TA; 5' Biotin modified universal primer 2 = CGC CAG GGT TTT CCC AGT CAC GAC
