# Supplementary material for: Evaluation of Methyl-Binding Domain Based Enrichment Approaches Revisited
Source: PLoS One. 2015 Jul 15;10(7):e0132205. doi: 10.1371/journal.pone.0132205 (PMC4503759; doi:10.1371/journal.pone.0132205)
Supplement: S3 Table — (DOCX) [file pone.0132205.s003.docx]

# S3 TABLE. Pyrosequencing assay design for sites detected by MethylCap.

| Chr | Location (bp) | Strand | Universal primer | PCR primers (forward and reverse primers are given on the top and bottom rows, respectively.) | Sequencing primers |
| --- | --- | --- | --- | --- | --- |
| 5 | 35938972 | + | 1 | AGG AAG TAG AGT GGG TGT GTAA | ATT TAG AGT TAG GGA AAA TG |
|  |  |  |  | GAC GGG ACA CCG CTG ATC GTT TAC CAC TAA CAT CA TAA CCT TTA TAT TTA TCC |  |
| 5 | 125254306 | - | 1 | TTG TTG TTG GTG TTT ATT ATT TGT TAA TT | GGT AAG TAG GAG GGG |
|  |  |  |  | GAC GGG ACA CCG CTG ATC GTT TAA AAA AAT CCT AAA CCT TCC AAA ACT TAT A |  |
| 7 | 13260310 | + | 1 | AGG TTT AGG GTG TTT TGT AAT TTA GGT A | GGT AGT AGG TTT ATT AAT AGG ATG |
|  |  |  |  | GAC GGG ACA CCG CTG ATC GTT TAC TCT TTT CTT AAC TTA TTA CCC CTA TCT |  |
| 7 | 37335819 | + | 1 | GGG AGG GTA GGT TTG AGT | ATA ATG AAA GTG GTG AGG |
|  |  |  |  | GAC GGG ACA CCG CTG ATC GTT TAT CAT ATC ATC CCT CAA CAT TAA ACC TCT CT |  |
| 17 | 7599547 | - | 1 | GGG AAG TAA AGT GAG TTA GTT AGA GAA T | GGA TTT TTA AAT TTT GGT GG |
|  |  |  |  | GAC GGG ACA CCG CTG ATC GTT TA ATAACAACTCTTTCTATAACCAAACATTC |  |
| 18 | 54175547 | - | 1 | AAT TTT AAG GTA TAA AGG AGA AAG AGT GAT | AGG AGA AAG AGT GAT TAG |
|  |  |  |  | GAC GGG ACA CCG CTG ATC GTT TAT CAT TTT AAA AAT CAA ATT CTC CCA CTA T |  |

Note. Chr is chromosome; 5' Biotin modified universal primer 1 = GAC GGG ACA CCG CTG ATC GTT TA; 5' Biotin modified universal primer 2 = CGC CAG GGT TTT CCC AGT CAC GAC
