# Supplementary material for: Evaluation of Methyl-Binding Domain Based Enrichment Approaches Revisited
Source: PLoS One. 2015 Jul 15;10(7):e0132205. doi: 10.1371/journal.pone.0132205 (PMC4503759; doi:10.1371/journal.pone.0132205)
Supplement: S4 Table — (DOCX) [file pone.0132205.s004.docx]

# S4 TABLE. Methylation detected by MethylMiner for CpG density range 1-3.

| Chromosome | Location (bp) | Sample ID | Technical Duplicate | % Methylation |
| --- | --- | --- | --- | --- |
| 3 | 88312855 | A | 1 | 87.93 |
| 3 | 88312855 | A | 2 | 87.77 |
| 3 | 88312855 | B | 1 | 86.25 |
| 3 | 88312855 | B | 2 | 85.15 |
| 4 | 121667807 | A | 1 | 68.82 |
| 4 | 121667807 | A | 2 | 71.34 |
| 4 | 121667807 | B | 1 | 69.28 |
| 4 | 121667807 | B | 2 | 68.87 |
| 7 | 117081770 | A | 1 | 68.12 |
| 7 | 117081770 | A | 2 | 70.53 |
| 7 | 117081770 | B | 1 | 70.32 |
| 7 | 117081770 | B | 2 | 72.47 |
| 9 | 115888715 | A | 1 | 93.41 |
| 9 | 115888715 | A | 2 | 94.11 |
| 9 | 115888715 | B | 1 | 93.91 |
| 9 | 115888715 | B | 2 | 93.85 |
| 10 | 94733369 | A | 1 | 97.36 |
| 10 | 94733369 | A | 2 | dispensation failure |
| 10 | 94733369 | B | 1 | 97.49 |
| 10 | 94733369 | B | 2 | 98.08 |
| 13 | 41715278 | A | 1 | 75.24 |
| 13 | 41715278 | A | 2 | 78.72 |
| 13 | 41715278 | B | 1 | 76.45 |
| 13 | 41715278 | B | 2 | 80.02 |
| 15 | 10987836 | A | 1 | 92.49 |
| 15 | 10987836 | A | 2 | 91.91 |
| 15 | 10987836 | B | 1 | 93.55 |
| 15 | 10987836 | B | 2 | 92.44 |
| 16 | 57553603 | A | 1 | 72.73 |
| 16 | 57553603 | A | 2 | 70.1 |
| 16 | 57553603 | B | 1 | 73.06 |
| 16 | 57553603 | B | 2 | 73.29 |
