# Supplementary material for: Evaluation of Methyl-Binding Domain Based Enrichment Approaches Revisited
Source: PLoS One. 2015 Jul 15;10(7):e0132205. doi: 10.1371/journal.pone.0132205 (PMC4503759; doi:10.1371/journal.pone.0132205)
Supplement: S5 Table — (DOCX) [file pone.0132205.s005.docx]

# S5 TABLE. Methylation detected by MethylCap for CpG density range 1-3.

| Chromosome | Location (bp) | Sample ID | Technical duplicate | % Methylation |
| --- | --- | --- | --- | --- |
| 5 | 35938972 | A | 1 | 62.67 |
| 5 | 35938972 | A | 2 | 59.15 |
| 5 | 35938972 | B | 1 | 57.73 |
| 5 | 35938972 | B | 2 | 52.84 |
| 17 | 7599547 | A | 1 | 91.34 |
| 17 | 7599547 | A | 2 | 89.80 |
| 17 | 7599547 | B | 1 | 88.84 |
| 17 | 7599547 | B | 2 | 95.71 |
| 18 | 54175547 | A | 1 | 76.68 |
| 18 | 54175547 | A | 2 | 72.79 |
| 18 | 54175547 | B | 1 | 63.08 |
| 18 | 54175547 | B | 2 | 68.11 |
