# Supplementary material for: Evaluation of Methyl-Binding Domain Based Enrichment Approaches Revisited
Source: PLoS One. 2015 Jul 15;10(7):e0132205. doi: 10.1371/journal.pone.0132205 (PMC4503759; doi:10.1371/journal.pone.0132205)
Supplement: S6 Table — (DOCX) [file pone.0132205.s006.docx]

# S6 TABLE. Methylation detected by MethylCap for CpG density range 9-11.

| Chromosome | Location (bp) | Sample ID | Technical duplicate | % Methylation |
| --- | --- | --- | --- | --- |
| 5 | 125254306 | A | 1 | 32.59 |
| 5 | 125254306 | A | 2 | 25.70 |
| 5 | 125254306 | B | 1 | 27.27 |
| 5 | 125254306 | B | 2 | 29.24 |
| 7 | 13260310 | A | 1 | 26.62 |
| 7 | 13260310 | A | 2 | 26.06 |
| 7 | 13260310 | B | 1 | 26.76 |
| 7 | 13260310 | B | 2 | 24.07 |
| 7 | 37335819 | A | 1 | 11.85 |
| 7 | 37335819 | A | 2 | 10.89 |
| 7 | 37335819 | B | 1 | 11.87 |
| 7 | 37335819 | B | 2 | 11.58 |
