# Supplementary material for: Evaluation of Methyl-Binding Domain Based Enrichment Approaches Revisited
Source: PLoS One. 2015 Jul 15;10(7):e0132205. doi: 10.1371/journal.pone.0132205 (PMC4503759; doi:10.1371/journal.pone.0132205)
Supplement: S1 Text — (DOCX) [file pone.0132205.s007.docx]

Supplementary material to:

**Evaluation of methyl-binding domain based enrichment approaches revisited**

Karolina A Aberg^1^, Linying Xie^1^, Robin F. Chan^1^, Min Zhao^1^, Ashutosh K. Pandey^2^, Gaurav Kumar^1^ , Shaunna L. Clark^1^, Edwin JCG van den Oord^1^

^1^ Center for Biomarker Research and Precision Medicine, Virginia Commonwealth University, Richmond, VA, USA

^2^ Center for Integrative and Translational Genomics and Department of Anatomy and Neurobiology, University of Tennessee Health Science Center, Memphis, TN, USA

METHODS FOR PREPARATION OF THE DBA/2J REFERNCE GENOME 2

Data collection for DBA/2J (D2J) mouse strain 2

Read alignment and variant calling 2

REFERECES 3

# METHODS FOR PREPARATION OF THE DBA/2J REFERNCE GENOME

## Data collection for DBA/2J (D2J) mouse strain

We downloaded the paired-end sequencing data for D2J mouse strain from the European Nucleotide Archive (ENA), accession number ERP000044[1] . ERP000044 sequencing data consists of 9 Illumina paired-end libraries sequenced on Illumina GAII platform. The read lengths for these libraries varies between 54-76 nt. We also downloaded Illumina paired-end sequencing data from SRA, accession number SRP001135[2]. This data consists of 3 libraries sequenced on Illumina GAII with read length of 100 nt.

## Read alignment and variant calling

Sequencing reads from each lane were trimmed off for the low quality bases and aligned to the C57BL/6J reference genome (mm10) using Burrows Wheeler Aligner[3] (version 6.1) and the parameters “-q 15”. Quality scores were recalibrated at the lane level using Genome Analysis Toolkit [4] (GATK version 2.7) ‘TableRecalibration’. All lanes from the same library were then merged together into a single BAM file using Picard tools (version 1.8, http://picard.sourceforge.net/). PCR duplicates were flagged at the library level using Picard ‘MarkDuplicates’. BAM files representing each library were merged together to create a single BAM file containing all the DBA/2J sequences. Finally, GATK ‘IndelRealigner’ was used to realign reads near indels from the Mouse Genome Project as well as potential Indels predicted by GATK. SNPs and Indels were identified using the GATK Unified Genotyper with default settings. In-house python scripts were used to filter low quality variants based on different criteria including strand bias, minimum mapping quality of reads and proximity to indels.

# REFERECES

1. Keane, T.M. *et al.* Mouse genomic variation and its effect on phenotypes and gene regulation. *Nature* **477**, 289-94 (2011).

2. Wang X, Agarwala R, Capra JA, Chen Z, Church DM, et al. (2010) High-throughtput sequenceing of the DBA/2J mouse genome. BMC Bioinformatics 11(Suppl 04): O7.

3. Li, H. & Durbin, R. Fast and accurate long-read alignment with Burrows-Wheeler transform. *Bioinformatics* **26**, 589-95 (2010).

4. McKenna, A. *et al.* The Genome Analysis Toolkit: a MapReduce framework for analyzing next-generation DNA sequencing data. *Genome Res* **20**, 1297-303 (2010).
